# Comparative Analysis of the Ginsenosides in *Panax vietnamensis* and Three *Panax* Species

**DOI:** 10.3390/molecules31101570

**Published:** 2026-05-08

**Authors:** Jiaxian Su, Kuntao Xu, Qimin Chen, Zhaosen Jia, You Deng, Mengjiao Zhu, Chongnan Wang, Lixia Zhang, Xiaojun Ma, Zuliang Luo

**Affiliations:** 1 State Key Laboratory for Quality Ensurance and Sustainable Use of Dao-Di Herbs, Institute of Medicinal Plant Development, Chinese Academy of Medical Sciences and Peking Union Medical College, Beijing 100193, China; 2 College of Pharmacy, Heilongjiang University of Chinese Medicine, Harbin 150040, China; 3 Yunnan Key Laboratory of Southern Medicine Utilization, Yunnan Branch, Institute of Medicinal Plant Development, Chinese Academy of Medical Sciences and Peking Union Medical College, Jinghong 666100, China

**Keywords:** *Panax vietnamensis*, *Panax* species, ginsenosides, qualitative identification, quantitative analysis

## Abstract

*Panax vietnamensis* Ha et Grushv. (Vietnamese ginseng) is a plant of the *Panax* genus, Araliaceae family. It is a rare medicinal plant found in China and Vietnam, known for its structurally diverse ginsenosides, and holds significant value in the pharmaceutical and health food sectors. As market demand and its value continue to rise, the *P. vietnamensis* industry has developed rapidly. However, since Vietnamese ginseng is difficult to distinguish from other *Panax* materials based on appearance, especially *Panax notoginseng*, there is a lack of relevant standards for quality control. In this study, UPLC-Q/TOF-MS technology was employed for the qualitative identification and comparative analysis of ginsenosides in different parts of *P. vietnamensis* and three other *Panax* species. Additionally, an UFLC-MS/MS method was established to determine the content of 21 ginsenosides in *P. vietnamensis*. Based on the UPLC-Q/TOF-MS analysis, 55 ginsenosides were preliminarily identified, including 30 protopanaxadiol-type, 21 protopanaxatriol-type, 3 ocotillol-type, and 1 oleanane-type ginsenosides. Further comparative analysis revealed variations in the ginsenosides of *P. vietnamensis* and three *Panax* species, identifying 41 components present in all species, while 14 saponins were detected only in some species. Compared to three *Panax* species, the main roots of *P. vietnamensis* contained characteristic components such as majonoside R_2_, majonoside R_1_, and vinaginsenoside R_2_. Quantitative analysis of 21 ginsenosides in different *Panax* species indicated that *P. vietnamensis* and *P. notoginseng* contained higher levels of ginsenoside Rg_1_, Rd, Rb_1_, and notoginsenoside R_1_. For instance, the average content of ginsenoside Rg_1_ in *P. vietnamensis* was 25.57 mg/g. Quantitative analysis of ginsenosides in different parts of *P. vietnamensis* revealed that the taproots and fibrous roots had a diverse array of ginsenosides with higher concentrations, while the stems and leaves contained fewer ginsenosides with lower levels. Significantly, certain components such as notoginsenoside Re and ginsenoside Rd, Re, and Rb2 were present at higher concentrations in the leaves. Based on the qualitative and quantitative analysis results of ginsenosides from different parts of *P. vietnamensis*, it was concluded that the main roots, fibrous roots, and leaves all have potential for development and utilization.

## 1. Introduction

The genus *Panax* L., belonging to the family Araliaceae under the order Apiales (Umbellales), is distributed across approximately 35 countries worldwide [1]. As important traditional medicinal herbs, plants of this genus are often used as adjunctive therapies for the prevention of diabetes, depression, cardiovascular diseases, and Alzheimer’s disease [2,3]. *Panax* species are significant as both medicinal and edible plants worldwide, holding substantial value in the health food and pharmaceutical industries. Representative species such as *Panax ginseng*, *Panax quinquefolius*, *P. notoginseng*, and *Panax japonicus* with their dried roots and rhizomes have been included in the Chinese Pharmacopoeia as early as 2015 [4,5].

**Figure 1:**
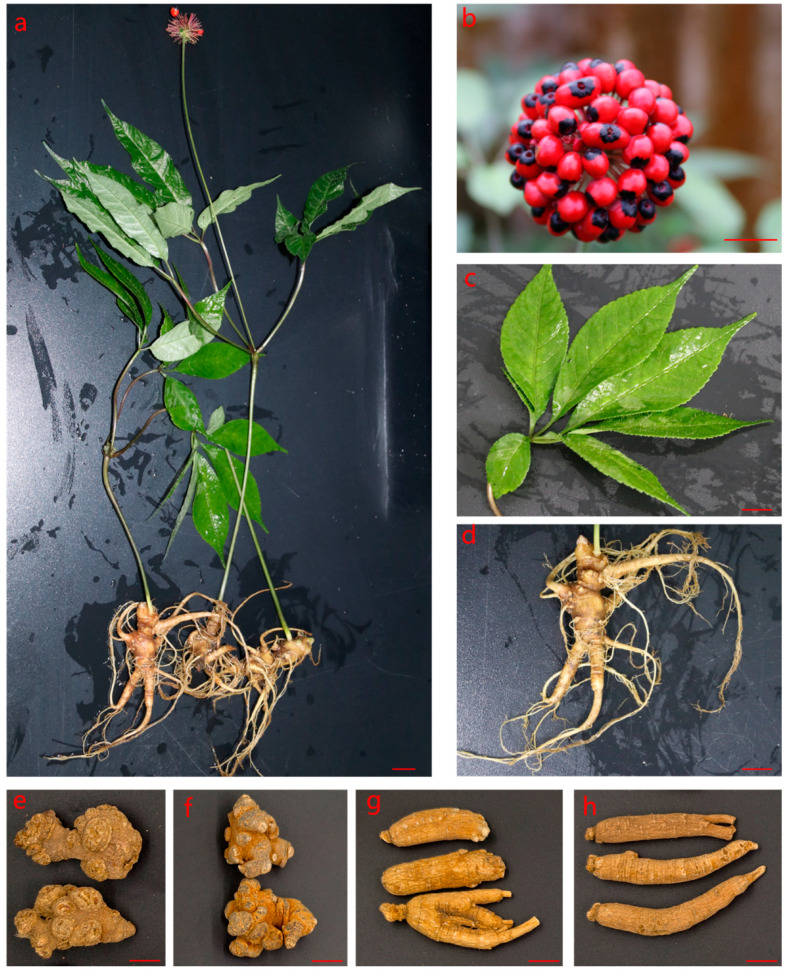
Morphology of *P. vietnamensis* and images of four *Panax* medicinal materials. The plant morphology of *P. vietnamensis* plants (**a**), *P. vietnamensis* fruits (**b**), *P. vietnamensis* leaves (**c**), underground part of *P. vietnamensis* (**d**), *P. vietnamensis* taproots (**e**), *P. notoginseng* taproots (**f**), *Panax ginseng* taproots (**g**), and *P. quinquefolius* taproots (**h**). The scale bar is 1 cm.

*Panax vietnamensis* Ha et Grushv., also known as Ngoc Linh ginseng or Jinping ginseng, is a perennial herbaceous plant belonging to the genus *Panax* of the Araliaceae family [6]. It was first discovered on Ngoc Linh Mountain in Kon Tum Province, Vietnam, in 1973. Subsequent studies have confirmed its natural distribution in the southern part of Yunnan Province, China [7]. In Vietnam, the distribution of *P. vietnamensis* is extremely restricted, mainly concentrated around Ngoc Linh Mountain and Hoang Lien Mountain in the central high-altitude areas, at an elevation ranging from 1700 to 2000 m [8]. The plants mostly grow in clusters, with a height ranging from 20 to 110 cm. The taproot is obconical or shortly fusiform, fleshy, and often branched. The rhizome grows in a twisted, nodular bamboo-like form underground, while the aerial stem is erect and solitary. There are three to six palmate compound leaves whorled at the top of the stem, and the leaflets are serrated. The flowers are bisexual or dioecious, arranged in a solitary terminal umbel, or two to several umbels clustered at the apex of the scape, with pale green corollas. The fruit is a drupe-like berry, oblate-reniform, red when mature, and usually marked with black spots at the apex. Each fruit contains multiple seeds, which are oval, triangular-ovate, or oblate in shape [1,8,9]. The morphological characteristics of *P. vietnamensis* are shown in Figure 1. In China, the distribution range of *P. vietnamensis* highly overlaps with the cultivation areas of *P. notoginseng*. Moreover, it bears a strong morphological resemblance to *Panax* species such as *P. ginseng* and *P. quinquefolius*, making its medicinal materials highly susceptible to adulteration and confusion.

As an important medicinal resource in the genus *Panax*, it is mainly harvested for its roots and rhizomes. It has long been regarded in folk medicine as a potent tonic herb, commonly used for postpartum recovery, as well as physical conditioning and vitality enhancement in the elderly and those debilitated by severe illness. In contrast, its aerial parts, including leaves and stems, are mostly utilized as an ingredient in herbal cooling teas [10]. Recorded in the Vietnamese Pharmacopoeia, this medicinal material is considered to have the effects of “greatly replenishing vital energy (Qi) and benefiting the lungs,” and is indicated for conditions such as “severe debility, lung deficiency with cough and dyspnea, and sore throat” [10,11]. Furthermore, in traditional Vietnamese folk medical practice, it is widely used for the adjuvant treatment of neurological and cardiovascular diseases, and is considered to possess anti-tumor, anti-aging, and stress-relieving properties [7]. Modern pharmacological studies have demonstrated that the ginsenosides from *P. vietnamensis* exert dynamic therapeutic effects, including anti-tumor, anti-inflammatory, hepatoprotective, renoprotective, neuroprotective, anxiolytic, anti-stress, and analgesic activities [1,12].

Studies have shown that a range of chemical components, including ginsenosides, volatile oils, and fatty acids, have been identified in different tissues of *P. vietnamensis* [13,14]. Among these, ginsenosides are considered the primary active constituents. Based on their skeletal structures, ginsenosides can be classified into two major categories: dammarane-type and oleanane-type (OA). The dammarane-type ginsenosides are further subdivided into three subtypes—protopanaxadiol (PPD), protopanaxatriol (PPT), and ocotillol (OT)—based on differences in the parent nucleus structure and substituent groups [15,16]. Like *P. ginseng*, *P. notoginseng*, and *P. quinquefolius*, *P. vietnamensis* contains PPD- and PPT-type ginsenosides, including ginsenosides Rb_1_, Rb_2_, Rc, Rd, Re, Rg_1_, and Rh_1_, notoginsenosides R_1_ and R_2_, and quinquenoside R_1_ [1,7,17,18]. The core competitiveness of *P. vietnamensis* lies in its unique OT-type ginsenosides, with an active substance content of up to 5.6%, accounting for over 50% of the total ginsenosides, thereby forming the basis of its distinctive pharmacological value [15,19]. Compared with other plants of the genus *Panax*, *P. vietnamensis* exhibits significant advantages in terms of total saponin and OT-type saponin content [19]. Current research on the chemical constituents of *P. vietnamensis* has primarily focused on its roots and rhizomes. According to the latest literature, more than 60 ginsenosides have been isolated and identified from *P. vietnamensis* to date [1,7].

Currently, systematic research on the component analysis and quality control of *P. vietnamensis* remains markedly insufficient. With advancements in analytical technology, ultra-high-performance liquid chromatography coupled with quadrupole time-of-flight mass spectrometry (UPLC-Q/TOF-MS) has become the mainstream technique for the identification and analysis of ginsenosides in *Panax* species [20,21,22,23,24]. This method combines the rapid separation capability of UPLC with the high resolution and sensitivity of quadrupole TOF-MS, making it suitable for the high-throughput identification of multiple ginsenosides in complex matrices. Therefore, this study aims to employ UPLC-Q/TOF-MS technology for the rapid analysis and identification of ginsenosides in *P. vietnamensis*, thereby providing an effective scientific basis for the identification, development, and utilization of *P. vietnamensis* as a medicinal resource. Furthermore, multi-component content analysis is an important reference for accurately reflecting and evaluating the quality of *P. vietnamensis*. While high-performance liquid chromatography equipped with a UV-VIS detector (HPLC-UV) has previously been used to detect various components such as ginsenoside Rd and ginsenoside Rc [25,26], LC-MS offers higher sensitivity than HPLC-UV, enabling the simultaneous and accurate quantification of multiple ginsenosides with weak ultraviolet absorption, thereby comprehensively reflecting the quality of the medicinal material. Currently, LC-MS has become the primary technique for the quantitative determination of ginsenosides [27,28,29]. In this study, using ginsenosides for which the reference standards are available as the assay indicators, we optimized the quantitative analysis conditions and ultimately established a UFLC-MS/MS method for the simultaneous determination of 21 ginsenosides. The established method was then used to comprehensively evaluate the differences in ginsenoside content between *P. vietnamensis* and three other *Panax* species, providing a scientific foundation for the development, utilization, and quality evaluation of *P. vietnamensis* as a medicinal resource.

## 2. Results and Discussion

### 2.1. Identification of Ginsenosides Using UPLC-Q/TOF-MS

**Figure 2:**
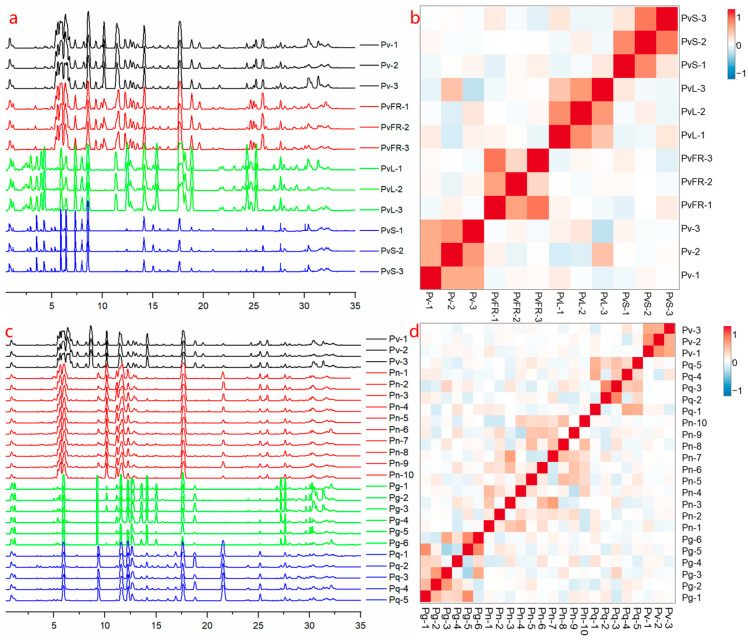
Comparative TIC chromatograms and correlation heatmaps. TIC chromatograms of different parts of *P. vietnamensis* (**a**); the correlation heatmap of different parts of *P. vietnamensis* (**b**); TIC chromatograms of the roots and rhizomes of four *Panax* species (**c**); and the correlation heatmap of the roots and rhizomes of four *Panax* species (**d**). Pv, *P. vietnamensis* taproots; PvFR, *P. vietnamensis* fibrous roots; PvL, *P. vietnamensis* leaves; PvS, *P. vietnamensis* stems; Pn, *P. notoginseng* taproots; Pg, *P. ginseng* taproots; and Pq, *P. quinquefolius* taproots.

According to the references, this experiment employed UPLC-Q/TOF-MS in negative-ion mode for sample analysis [20,21,22,23,24], and raw data were processed using Progenesis QI V2.0 software. Subsequently, online software Metaboanalyst 6.0 (https://www.metaboanalyst.ca/; accessed on 24 March 2026) was used for sample correlation analysis. The total ion current (TIC) chromatograms and correlation analysis results for the four *Panax* species and different parts of *P. vietnamensis* are shown in Figure 2. From the TIC chromatograms and correlation analysis plots, it can be observed that there are significant differences in the chemical composition among different parts (taproot, fibrous root, stem, and leaf) of *P. vietnamensis*, as well as among the taproots of the four *Panax* species. These inter-group differences likely reflect intrinsic distinctions in the chemical profiles of the samples, warranting further systematic component analysis of samples from different sources.

In this study, the literature on mass spectrometry analysis of ginsenosides in *Panax* plants was systematically integrated to perform qualitative identification of the components. In negative-ion mode, ginsenosides typically exhibit [M−H]^−^ and [M+HCOO]^−^ ion peaks, corresponding to deprotonated molecular ions and formate adduct ions, respectively. This phenomenon arises from the addition of a certain concentration of formic acid to the mobile phase, which facilitates the formation of adduct ions between formate and the target compounds. Using [M−H]^−^ as precursor ions, their fragment information was elucidated via MS/MS analysis. Combined with data from the literature and the MS/MS fragment information on reference standards, the chromatographic peaks separated by UPLC were analyzed and identified one by one, and their structural formulas and molecular formulas were deduced. The detailed analysis results are presented in Table 1. Ultimately, a total of 55 ginsenosides were tentatively identified, including 30 PPD-type, 21 PPT-type, 3 OT-type, and 1 OA-type saponin.

**Figure 3:**
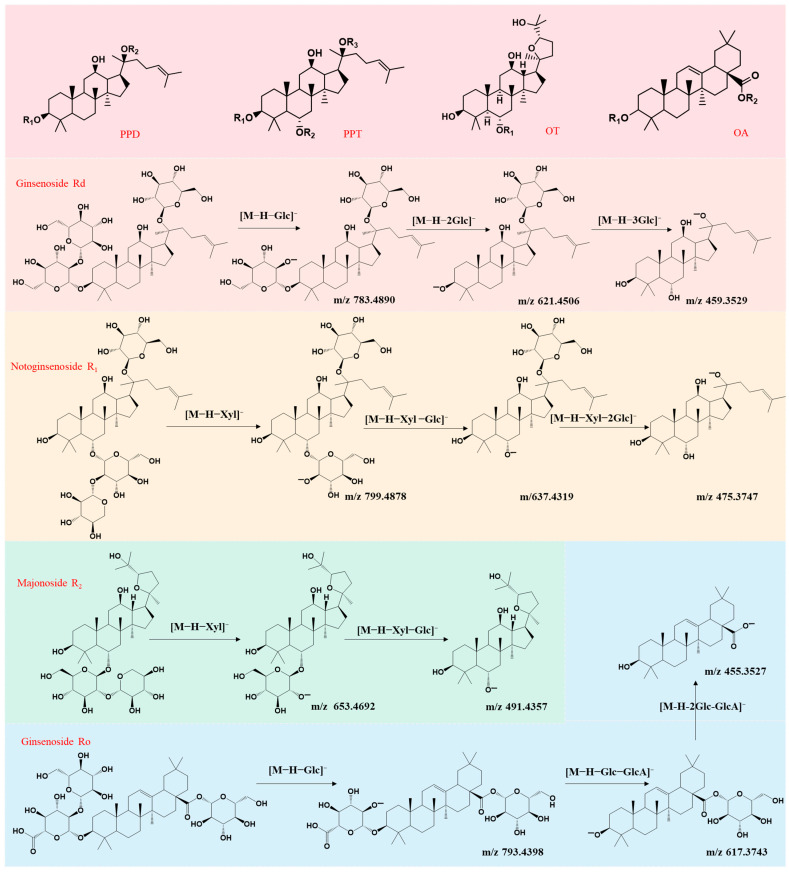
Schematic diagrams of the skeletal structure of four types of ginsenosides and fragmentation pathways of representative components. PPD, protopanaxadiol-type; PPT, protopanaxatriol-type; PPT, protopanaxatriol-type; PPT, protopanaxatriol-type; Glc, glucose; Xyl, xylose; and GlcA, glucuronic acid.

In MS/MS spectra, these compounds readily undergo glycosidic bond cleavage, with common deglycosylated fragment ions such as [M+HCOO-162]^−^ or [M−H−162]^−^ (loss of one glycosyl group at *m*/*z* 162), and [M+HCOO-324]^−^ or [M−H-324]^−^ (loss of two glycosyl groups at *m*/*z* 324). The characteristic fragment ions for PPD-, PPT-, OT-, and OA-type ginsenosides are *m*/*z* 459, 475, 491, and 455, respectively. The structures of these four saponin types are shown in Figure 3. By analyzing these fragment ions and their accurate masses, the structural types of ginsenosides can be deduced, while the mass losses further facilitate the identification of the glycosyl types. A schematic diagram of the fragmentation pathways for representative ginsenosides is presented in Figure 3.

The detailed analysis and identification process for PPD-type ginsenosides was as follows: compound 41 had a retention time of 17.57 min. In the MS spectrum, its quasi-molecular ion peak [M−H]^−^ at *m*/*z* 945.5476 was observed, along with an adduct ion peak [M+HCOO]^−^ at *m*/*z* 991.5468. MS/MS analysis revealed characteristic fragment ions: *m*/*z* 783.4890 corresponding to [M−H−Glc]^−^; another fragment ion at *m*/*z* 621.4506 corresponding to [M−H−2Glc]^−^; and *m*/*z* 459.3529 corresponding to the aglycone. This compound exhibited the typical fragmentation behavior of PPD-type ginsenosides. By comparing with data from the literature, the retention times of reference standards, and high-resolution mass spectrometry information, compound 41 was identified as ginsenoside Rd [30]. Compound 42 had a retention time of 18.81 min. The MS spectrum showed a quasi-molecular ion peak [M−H]^−^ at *m*/*z* 987.5548 and an adduct ion peak [M+HCOO]^−^ at *m*/*z* 1033.5546. In the MS/MS spectrum, characteristic fragment ions included *m*/*z* 945.4710 as [M−H−Acetyl]^−^, *m*/*z* 783.4519 as [M−H−Acetyl−Glc]^−^, *m*/*z* 621.4506 as [M−H−Acetyl−2Glc]^−^, and *m*/*z* 459.3529 as the aglycone. Based on reports from the literature, compound 42 was identified as pseudoginsenoside Rc_1_ [32]. Using the same approach, a total of 30 PPD-type ginsenosides were identified, including ginsenoside Rb_1_, ginsenoside Rc, ginsenoside Rb_3_, ginsenoside F_2_, quinquenoside R_1_, and vinaginsenoside R_8_. During the collision-induced dissociation of PPD-type ginsenosides, in addition to the characteristic fragment ions produced by the loss of sugar chains, a specific signal peak at *m*/*z* 459 corresponding to the aglycone was also observed. This peak serves as a hallmark fragmentation product of the PPD-type ginsenosides.

The detailed analysis and identification process for PPT-type ginsenosides was as follows: compound 5, with a retention time of 5.59 min in the TIC chromatogram, showed two key signals in the MS spectrum: a quasi-molecular ion peak [M−H]^−^ at *m*/*z* 931.5308 and an adduct ion peak [M+HCOO]^−^ at *m*/*z* 977.5345. MS/MS fragmentation yielded three characteristic fragment ions: 799.4878 ([M−H−Xyl]^−^), 637.4319 ([M−H−Xyl−Glc]^−^), and 475.3747 ([M−H-Xyl−2Glc]^−^), with the ion at *m*/*z* 475.3747 identified as the aglycone. Based on the relevant literature, the retention times of reference standards, and mass spectrometry data, compound 5 was identified as notoginsenoside R_1_ [30]. Compound 22, with a retention time of 10.16 min, exhibited a quasi-molecular ion peak [M−H]^−^ at *m*/*z* 769.4743 and an adduct ion peak [M+HCOO]^−^ at *m*/*z* 815.4790 in the MS spectrum. In the MS/MS spectrum, the characteristic fragment ion at *m*/*z* 637.4318 corresponded to [M−H−Ara]^−^, and the ion at *m*/*z* 475.4190 corresponded to [M−H−Ara−Glc]^−^. According to the data available in the literature, the mass spectral characteristics of compound 22 were highly consistent with those of the ginsenoside F_5_ [30]. Using the same approach, a total of 21 PPT-type ginsenosides were identified, including ginsenoside Re, Rg_1_, Rg_2_, Rh_1_, and pseudoginsenoside F_11_. Mass spectrometry analysis indicated that, in addition to fragment signals from the loss of sugar chains, PPT-type ginsenosides also exhibited a characteristic ion peak at *m*/*z* 475 in the MS/MS spectra, which was identified as the characteristic aglycone fragment of PPT-type ginsenosides.

The detailed analysis and identification process for OT-type ginsenosides was as follows: compound 9, with a retention time of 6.38 min in the TIC chromatogram, produced a quasi-molecular ion peak [M−H]^−^ at *m*/*z* 785.4654 and an adduct ion peak [M+HCOO]^−^ at *m*/*z* 831.4688 in the MS spectrum. In the MS/MS spectrum, the characteristic fragment ion at *m*/*z* 653.4692 corresponded to [M−H−Xyl]^−^, and the fragment ion at *m*/*z* 491.4357 corresponded to [M−H−Xyl−Glc]^−^, which was the aglycone. Based on reports from the literature, compound 9 was identified as majonoside R_2_ [31]. Using the same method, a total of two OT-type ginsenosides were identified, namely majonoside R_1_ and pseudoginsenoside Rt_4_. In the MS/MS spectra, besides the fragment ions resulting from sugar chain cleavage, these ginsenosides also exhibited a characteristic fragment ion at *m*/*z* 491 specific to OT-type ginsenosides, corresponding to their aglycone structure.

Furthermore, the OA-type ginsenoside Ro was identified. In the MS spectrum, this compound exhibited a quasi-molecular ion peak [M−H]^−^ at *m*/*z* 955.4940 and an adduct ion peak [M+HCOO]^−^ at *m*/*z* 1001.4919. MS/MS analysis revealed characteristic fragment ions: *m*/*z* 793.4398 corresponding to [M−H−Glc]^−^, *m*/*z* 617.3743 corresponding to [M−H−Glc−GlcA]^−^, and *m*/*z* 455.3527 derived from [M−H−2Glc−GlcA]^−^, representing its aglycone.

Due to the limited availability of reference standards and data from the literature, the structures of 10 isomers among the 55 tentatively identified compounds require further confirmation. In addition, some compounds with undetermined structures remain in the samples, which need to be identified through subsequent isolation, purification, and structural elucidation. Furthermore, statistical analysis of the detection of the 55 ginsenosides in the samples of four *Panax* species and different parts of *P. vietnamensis* revealed that 41 components were detected in all samples, while 14 components were detected only in some sample groups. The detailed results are shown in Supplementary Table S1. These components may serve as potential quality markers for medicinal materials from *Panax* species in future studies and can be developed for quality control.

### 2.2. Development and Validation of a UFLC-QTRAP-MS/MS Method for 21 Ginsenosides

#### 2.2.1. Optimization of UFLC-QTRAP-MS/MS Condition

**Figure 4:**
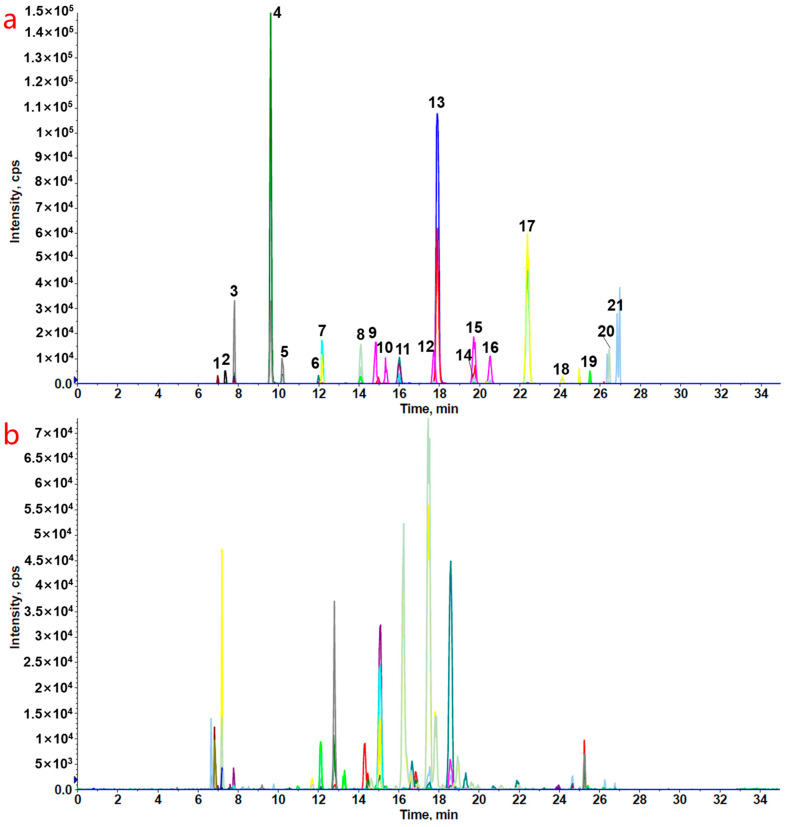
MRM chromatograms of mixed standard solution (**a**) and *P. vietnamensis* (**b**) in negative-ion mode. Peak 1: notoginsenoside R_1_; 2: ginsenoside Re; 3: ginsenoside Rg_1_; 4: vinaginsenoside R_8_; 5: vinaginsenoside R_4_; 6: pseudoginsenoside F_11_; 7: ginsenoside Rf; 8: notoginsenoside Fa; 9: ginsenoside Rg_2_; 10: ginsenoside Rh_1_; 11: ginsenoside Rb_1_; 12: ginsenoside Rc; 13: ginsenoside Ro; 14: ginsenoside Rb_2_; 15: ginsenoside F_1_; 16: ginsenoside Rb_3_; 17: quinquenoside R_1_; 18: ginsenoside Rd; 19: notoginsenoside Fe; 20: ginsenoside F_2_; and 21: ginsenoside Rg_3_.

To obtain the optimal mass spectrometric conditions for the quantitative analysis of ginsenosides, systematic optimization of the analytical parameters was performed. A standard solution at 100 ng/mL was prepared using a mixed solvent of acetonitrile and water (1:1, *v*/*v*) and directly infused into the mass spectrometer at a constant flow rate of 10 μL/min via a syringe pump. During mass spectrometric analysis, full-scan mode was first applied to acquire positive- and negative-ion signals separately. The optimal ionization mode was selected based on response intensity and the signal-to-noise ratio. Subsequently, the quasi-molecular ions of the target compounds were designated as precursor ions. Characteristic product ions were screened via MS2 scanning, and parameters such as the collision energy were systematically optimized. Finally, quantitative ion transitions with high sensitivity and superior specificity, together with the corresponding mass spectral conditions, were established. The characteristic product ion exhibiting the highest signal intensity was chosen for the quantitative transition. The optimized ion transitions and relevant parameters are listed in Supplementary Table S2. As a core parameter in liquid chromatography, the composition of the mobile phase directly affects the ionization efficiency and chromatographic separation of analytes. In this study, the optimal mobile phase composition and other chromatographic conditions were determined with reference to the published literature [28]. Under the optimized mass spectrometric and chromatographic conditions, the extracted ion chromatogram of the target analyte is shown in Supplementary Figure S1, and the typical multiple reaction monitoring (MRM) chromatograms of the target analytes are presented in Figure 4.

#### 2.2.2. Method Validation

Experimental data indicated (Supplementary Table S3) that all 21 ginsenosides exhibited excellent linear correlations within the set concentration range, with correlation coefficients (*r*) greater than 0.999. The limits of detection (LOD) and limits of quantification (LOQ) were below 0.5 μg/mL and 1 μg/mL, respectively. The intra-day precision presented relative standard deviation (RSD) values ranging from 3.06% to 5.28%, and the inter-day precision RSD values ranged from 2.33% to 5.43%. The recoveries of the 21 target components were between 79.22% and 112.77%, with RSDs of 2.02–8.22%. After the sample solution was stored at room temperature and determined every 12 h within 48 h, the RSD values of the peak areas for all analytes were less than 2.48%, demonstrating satisfactory sample stability. The RSD of the repeatability test was lower than 5.43%. These results fully verified that the established method possesses high sensitivity, favorable stability, and reliable repeatability, and is suitable for the simultaneous quantitative determination of the 21 ginsenosides in *Panax* samples.

#### 2.2.3. Sample Analysis

**Figure 5:**
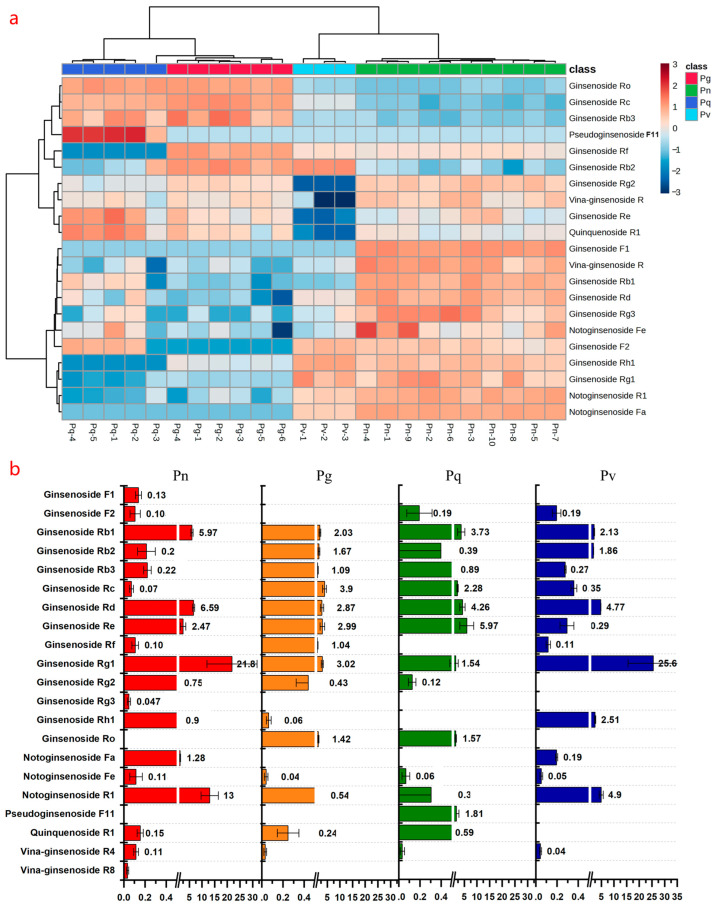
Heatmap analysis of the content determination results of ginsenosides in *P. vietnamensis* and three *Panax* species. The cluster heatmap (**a**), the bar chart of contents (**b**). Pv, *P. vietnamensis* taproots; Pn, *P. notoginseng* taproots; Pg, *P. ginseng* taproots; and Pq, *P. quinquefolius* taproots.

A method was established for the determination of 21 ginsenosides in different parts of *P. vietnamensis* and in multiple batches of *P. ginseng*, *P. notoginseng*, and *P. quinquefolius* samples. The target analytes were identified by retention time and characteristic ion pairs, and quantitative analysis was performed using the external standard method. The sample determination results are shown in Supplementary Table S4. Subsequently, the online software Metaboanalyst 6.0 was used for heatmaps and ANOVA analysis. The experimental results indicated that the contents of the 21 ginsenosides varied significantly among samples from different sources. The cluster heatmap of ginsenosides from four *Panax* species (*P. vietnamensis*, *P. ginseng*, *P. quinquefolius*, and *P. notoginseng*) is shown in Figure 5. The saponin contents of *P. vietnamensis* and *P. notoginseng* showed high similarity, clustering into one major group, while *P. ginseng* and *P. quinquefolius* exhibited high similarity, clustering into another major group. In *P. vietnamensis* and *P. notoginseng*, the contents of ginsenoside Rg_1_, Rd, and notoginsenoside R_1_ were higher. ANOVA analysis further indicates significant content differences in saponins among different species. For example, the average content of ginsenoside Rg_1_ in *P. vietnamensis* was 25.57 mg/g, compared to 3.02 mg/g in *P. ginseng*, 1.54 mg/g in *P. quinquefolius*, and 21.83 mg/g in *P. notoginseng*. On the other hand, *P. ginseng* and *P. quinquefolius* showed high similarity; as well as ginsenoside Rd and Rb_1_, the main components included ginsenoside Rc, Ro, and Re.

In addition, we compared the quantitative results of saponin components in four *Panax* species with those reported in the relevant literature. The results showed that the most abundant saponin components in *P. notoginseng* were ginsenoside Rg_1_, notoginsenoside R_1_, and ginsenoside Rb_1_, which is generally consistent with the previous literature [35,36]. However, the literature reported that the content of ginsenoside Rb_1_ could reach 20 mg/g, whereas the measured values in this study were relatively lower. The saponin contents in *P. ginseng* were consistent with reports, with the major components being ginsenoside Rg_1_, Rb_1_, Rc, and Re [37,38]. For *P. quinquefolius*, the components with relatively high contents included ginsenoside Rb_1_ and Re [39].

The determination results of different parts of *P. vietnamensis* showed that the content and variety of ginsenosides quantitatively detected in the taproots and fibrous roots were greater (Supplementary Figure S2). For example, the content of ginsenoside Rg_1_ in the taproots and fibrous roots was 25.57 and 14.81 mg/g, respectively, while it was 3.16 mg/g in the leaves and not detected in the stems. Notably, the content of ginsenoside Rd in the leaves reached as high as 8.62 mg/g, which was higher than that in the taproots (4.77 mg/g), fibrous roots (5.41 mg/g), and stems (1.34 mg/g). Moreover, the contents of notoginsenoside Fe, ginsenoside F_2_, ginsenoside Rb_2_, and ginsenoside Re in the leaves of *P. vietnamensis* were all higher than those in other parts. ANOVA analysis further indicates significant content differences in saponins among different parts of *P. vietnamensis*.

Based on ginsenosides’ quantitative results in different parts of *P. vietnamensis*, from the perspective of resource development, in addition to the taproots, the fibrous roots and leaves also possess value for development and utilization. For the quality evaluation of *P. vietnamensis* as a medicinal material, the ginsenosides with relatively high contents, such as ginsenoside Rg_1_, ginsenoside Rd, notoginsenoside R_1_, ginsenoside Rb_1_, and ginsenoside Rh_1_, can all serve as evaluation indicators. However, the established method for determining the 21 ginsenosides in this study did not include the characteristic marker components of *P. vietnamensis*, namely majonoside R_1_, majonoside R_2_, and vinaginsenoside R_2_. Therefore, the content determination method should be further improved in the future when the reference standards become available.

## 3. Materials and Methods

### 3.1. Plant Materials, Chemicals, and Reagents

Three batches of three-year-old *P. vietnamensis* samples were collected from the experimental plantation in Jinping County, Yunnan Province. They were identified by Professor Ma Xiaojun from the Institute of Medicinal Plants, Chinese Academy of Medical Sciences as *Panax vietnamensis* Ha et Grushv., a plant of the *Panax* species in the Araliaceae family. Actual specimens of *P. vietnamensis* plants and their different parts are shown in Figure 1. After bringing the plant materials to the laboratory, the soil was rinsed off the roots, then the plant tissues were separated into taproots, fibrous roots, leaves, and stems, and they were dried at 60 °C in a constant temperature drying oven until reaching a constant weight. The dried samples were then crushed, homogenized, and stored in Ziplock bags at 4 °C until analysis. In addition, 6 batches of ginseng samples (the roots and rhizomes of *P. ginseng*), 5 batches of American ginseng samples (the roots and rhizomes of *P. quinquefolius*), and 10 batches of Sanqi samples (the roots and rhizomes of *P. notoginseng*) were collected from Hehuachi TCM market (Chengdu, China), Anguo TCM market (Anguo, China), and Bozhou TCM market (Bozhou, China). All sample information of the plant materials can be found in Supplementary Table S5.

Twenty-eight reference standards, including for ginsenoside Re, Rg_1_, Rg_2_, Rh_1_, Rb_1_, Ro, Rc, Rb_3_, Rd, F_1_, and F_2_, etc., were purchased from Chengdu Must Biotechnology Co., Ltd. (Chengdu, China). Detailed information on reference standards can be found in Supplementary Table S6. HPLC-grade formic acid, acetonitrile, and methanol came from Fisher Scientific. Other reagents and chemicals were sourced from Sinopharm Chemical Reagent Beijing Co., Ltd. (Beijing, China). Ultrapure water for HPLC-MS/MS analysis was prepared using a Milli-Q system.

### 3.2. Instruments

For qualitative analysis, we employed an Acquity UPLC H-Class system, paired with a Xevo G2-S QTof™ mass spectrometer, both from Waters in Milford, MA, USA. For quantitative assessments, a Shimadzu ultra-fast liquid chromatography (UFLC) system, linked to an Applied Biosystem Sciex 5500 QTRAP^®^ mass spectrometer (Framingham, MA 01701, USA), was used to ensure accurate measurements. Sample and mobile phase preparations were meticulously carried out using a range of pieces of equipment, including a KQ-400DE ultrasonic cleaner from Kunshan Ultrasonic Instrument Co., Ltd. (Kunshan, China), a GZX-9070MBE electric blast drying oven supplied by Shanghai Boxun Industrial Co., Ltd. (Shanghai, China), a Milli-Q purification system from Millipore, and an electric blast drying oven from Shanghai Boxun Industrial Co., Ltd. (Shanghai, China). Electronic analytical balances from Sartorius Scientific Instruments were also utilized for precise weighing.

### 3.3. Standard and Sample Solution Preparation

First, 28 ginsenoside stock solutions were made separately, each with a 0.1 mg/mL concentration in methanol. Next, these standard stock solutions were diluted with a methanol–water mixture (70:30, *v*/*v*) to form mixed standard working solutions. All solutions were stored at 4 °C in a fridge for later use. After that, six different concentrations (20, 10, 5, 2, 1, and 0.5 µg/mL) were analyzed, and calibration curves were created by plotting the peak area versus the analyte concentration.

In the chemical composition analysis, meticulous procedures were employed. First, 1.0 g of homogenized plant materials was precisely weighed. These samples then underwent ultrasonic extraction at a frequency of 40 kHz, utilizing 20 mL of a methanol–water mixture (70:30, *v*/*v*) for a duration of 60 min. Following extraction, 1.0 mL of the upper layer of the solution was carefully filtered through a 0.22 µm syringe nylon filter. The filtered solution was then transferred to an injection bottle for subsequent analysis using a UPLC-Q/TOF-MS system. For the quantitative analysis of ginsenosides in *Panax* species plant materials, similar steps were taken. Homogenized samples (1.0 g) were accurately weighed and subjected to ultrasonic extraction at 40 kHz with 20 mL of the same methanol–water mixture for 60 min. After extraction, the solution was allowed to stand and cool, with the original solvent weight restored by adding more extract solvent. Subsequently, 1.0 mL of the extract was diluted to 5 mL, mixed thoroughly for 30 s, and filtered. All processed samples were stored in a refrigerator at 4 °C until analysis.

### 3.4. Ginsenoside Analysis Using UPLC-Q/TOF-MS/MS

To accurately identify saponins in plant materials from the *Panax* species, a sophisticated analytical approach was employed. The Acquity UPLC H-Class system, in tandem with a Xevo G2-S QTof™ mass spectrometer from Waters in Milford, MA, USA, was used for this precise task. The chromatographic separation was carried out on an ACQUITY UPLC™ HSS T3 column (100 mm × 2.1 mm, 1.8 μm) and kept at a constant temperature of 35 °C. A flow rate of 0.3 mL/min was maintained, and samples were injected in a volume of 1.0 μL. The mobile phase comprised water containing 0.1% formic acid (A) and acetonitrile (B), employing a gradient elution: 90–70% (A) from 0 to 5 min; 70–67% (A) from 5 to 10 min; 67–64% (A) from 10 to 22 min; 64–15% (A) from 22 to 28 min; 15–5% (A) from 28 to 29 min; maintaining at 5% A from 29 to 34 min; 5–90% (A) from 34 to 34.5 min; and then equilibration at 90% (A) to 40 min.

Negative mode electrospray ionization mass spectrometry was employed. The mass spectrometer was precisely configured: a capillary voltage of 2.5 kV, sampling and extraction cones at 40 V and 4.0 V, respectively, a source temperature of 100 °C, and a desolvation temperature of 250 °C. For MS acquisition, a collision energy of 6 eV was used, and 45 eV for MSE acquisition. The cone gas flow rate was kept at 50 L/h. TOF-MS scanning spanned *m*/*z* 100–1500 Da, with data collected via MassLynx V4.1 software.

### 3.5. Quantitative Analysis of 21 Ginsenosides Using UFLC-QTRAP-MS/MS

#### 3.5.1. UFLC-QTRAP-MS/MS Conditions

To conduct a precise quantitative analysis, we employed a sophisticated instrumental setup. A Shimadzu ultra-fast liquid chromatography (UFLC) system was paired with an Applied Biosystem Sciex 5500 QTRAP^®^ mass spectrometer via an ESI interface. This combination ensured high-resolution separation and the sensitive detection of analytes. Chromatographic separation was carried out on an ACQUITY UPLC™ HSS T3 column, which provided an excellent peak shape and retention time reproducibility. The mobile phase consisted of water with 0.1% formic acid (A) and acetonitrile (B), with the following gradient elution: 90–70% (A) from 0 to 5 min; 70–67% (A) from 5 to 10 min; 67–64% (A) from 10 to 22 min; 64–15% (A) from 22 to 28 min; 15–5% (A) from 28 to 29 min; maintaining at 5% A from 29 to 31 min; 5–90% (A) from 31 to 31.5 min; and then equilibration at 90% (A) to 35 min. The column was operated at a flow rate of 0.3 mL/min, and the injection volume was 5.0 μL.

For mass spectrometry analysis, we utilized an electrospray ionization (ESI) source operating in negative mode. Precise settings were applied: curtain gas at 35 psi, nebulizer gas at 50 psi, and auxiliary gas also at 50 psi. The ion spray voltage was adjusted to −4500 V, while the source temperature was kept at 550 °C to ensure optimal ionization conditions. We employed multiple reaction monitoring (MRM) for accurate quantitation, with each MRM transition having a dwell time of 50 ms. Specific potentials were set for declustering (−160 V), entrance (−10 V), and collision cell exit (−15 V). The UFLC-QTRAP-MS/MS system was controlled, and data were acquired and processed using Applied Biosystems Analyst software, version 1.6.

#### 3.5.2. Validation Protocol

In strict adherence to the International Conference on Harmonization (ICH) guidelines for analytical method validation, a comprehensive assessment was conducted of the analytical method [40]. This evaluation encompassed several key parameters: linearity, sensitivity, precision (both intra-day and inter-day), stability, repeatability, and accuracy. Calibration curves were constructed by plotting the analyte concentration (*X*) against the peak area (*Y*), analyzing at least six concentration levels in triplicate. The limits of detection (LOD) and quantification (LOQ) were determined using signal-to-noise ratios of about 3 and 10, respectively. Precision was gauged through repeated analyses of standard samples, both within a single day and across three consecutive days. Accuracy was verified by adding target analytes to previously analyzed samples, while stability was monitored by analyzing sample solutions at room temperature every 12 h over a two-day period. Finally, repeatability was ensured by preparing and analyzing six independent sample solutions.

## 4. Conclusions

This study established an analytical method for ginsenosides in *P. vietnamensis* and three other *Panax* species based on UPLC-Q/TOF-MS. A total of 55 ginsenosides were tentatively identified, including 30 PPD-type, 21 PPT-type, 3 OT-type, and 1 OA-type saponins. Furthermore, from a metabolomics perspective, the changes in the chemical components of *P. vietnamensis* and three *Panax* species were analyzed. The results showed that 41 components were detected in all samples, while 14 ginsenosides were detected only in some groups. The taproots of *P. vietnamensis* contained characteristic components such as majonoside R_2_, majonoside R_1_, and vinaginsenoside R_2_, which can serve as key indicator components for the identification of *P. vietnamensis* in the future.

In addition, the content determination results showed that ginsenosides in *P. vietnamensis* and *P. notoginseng* had high similarity, with higher contents of ginsenoside Rg_1_, ginsenoside Rd, notoginsenoside R_1_, and ginsenoside Rb_1_ in *P. vietnamensis* and *P. notoginseng*. For different parts of *P. vietnamensis* samples, the taproots and fibrous roots were rich in various ginsenosides in high contents, while the contents of notoginsenoside Re, ginsenoside Rd, ginsenoside Re, and ginsenoside Rb_2_ in the leaves were higher than those in the taproots. Based on the quantitative analysis results of different parts of *P. vietnamensis*, from the perspective of resource development, in addition to the taproots, the fibrous roots and leaves also possessed value for development and utilization. For a quality evaluation of *P. vietnamensis* as a medicinal material, the ginsenosides with relatively high contents, such as ginsenoside Rg_1_, ginsenoside Rd, notoginsenoside R_1_, ginsenoside Rb_1_, and ginsenoside Rh1, can all serve as evaluation indicators. Combined with the results of this study, comprehensive research on sustainable resource utilization should be strengthened in the future to achieve the efficient utilization of the whole *P. vietnamensis* plant, address the issue of resource endangerment, and ensure the healthy development of the industry.

## Figures and Tables

**Table 1:** Ginsenosides identified using UPLC-Q-TOF-MS.

No.	Compound	Skeletal Structure	Formula	Rt/min	Adducts	Expected (*m*/*z*)	Measured (*m*/*z*)	Mass Error/ppm	Fragment Ion (*m*/*z*)	Reference
1	Floralginsenoside A	PPT	C_42_H_72_O_16_	4.5	[M+HCOO]^−^	877.4797	877.4805	0.92	831.4778 [M−H]^−^; 651.4104 [M−H-Glc]^−^	[30]
2	6′-Acetyl-ginsenoside F_1_	PPT	C_38_H_64_O_10_	4.99	[M+HCOO]^−^	725.4476	725.4478	0.28	679.4439 [M−H]^−^	[30]
3	20-O-Glucoginsenoside Rf	PPT	C_48_H_82_O_19_	5.13	[M+HCOO]^−^	1007.5427	1007.5458	3.10	961.5408 [M−H]^−^; 799.4835 [M−H-Glc]^−^	[30]
4	20-O-Glucoginsenoside Rf isomer	PPT	C_48_H_82_O_19_	5.4	[M+HCOO]^−^	1007.5427	1007.5458	3.10	961.5408 [M−H]^−^; 799.4835 [M−H-Glc]^−^	[30]
5 *	Notoginsenoside R_1_	PPT	C_47_H_80_O_18_	5.59	[M+HCOO]^−^	977.5321	977.5345	2.43	931.5308 [M−H]^−^; 799.4878 [M−H-Xyl]^−^	[30]
6	Majonoside R_1_	OT	C_42_H_72_O_15_	5.79	[M+HCOO]^−^	861.4848	861.4861	1.54	815.4835 [M−H]^−^; 653.4290 [M−H-(Glc-H2O) ]^−^	[30]
7 *	Ginsenoside Re	PPT	C_48_H_82_O_18_	5.79	[M+HCOO]^−^	991.5478	991.5468	−0.98	945.5476 [M−H]^−^	[30]
8 *	Ginsenoside Rg_1_	PPT	C_42_H_72_O_14_	5.98	[M+HCOO]^−^	845.4899	845.4913	1.70	799.4872 [M−H]^−^; 637.4328 [M−H-Glc]^−^; 475.3786 [M−H-2Glc]^−^	[30]
9	Majonoside R_2_	OT	C_41_H_70_O_14_	6.38	[M+HCOO]^−^	831.4742	831.4688	−6.51	785.4654 [M−H]^−^; 653.4692 [M−H-Xyl]^−^	[31]
10	Pseudoginsenoside Rs_1_	PPT	C_51_H_84_O_21_	6.47	[M+HCOO]^−^	1033.5583	1033.5632	4.71	987.5548 [M−H]^−^	[32]
11	Ginsenoside M_7cd_	PPT	C_36_H_62_O_10_	6.51	[M+HCOO]^−^	699.4320	699.4297	−3.22	653.3382 [M−H]^−^	[30]
12	Ginsenoside III	PPD	C_48_H_80_O_19_	6.67	[M+HCOO]^−^	1005.5270	1005.5277	0.67	959.5258 [M−H]^−^	[30]
13 *	Vinaginsenoside R_8_	PPD	C_48_H_82_O_19_	7.3	[M+HCOO]^−^	1007.5427	1007.5458	3.10	961.5408 [M−H]^−^; 799.4835 [M−H-Glc]^−^	[30]
14	Malonyl-ginsenoside Re_1_	PPT	C_51_H_84_O_21_	7.3	[M+HCOO]^−^	1033.5583	1033.5546	−3.61	987.5548 [M−H]^−^	[32]
15 *	Vinaginsenoside R_4_	PPT	C_48_H_82_O_19_	7.76	[M+HCOO]^−^	1007.5427	1007.5458	3.10	961.5408 [M−H]^−^; 799.4835 [M−H-Glc]^−^	[30]
16	Ginsenoside Rs_3_	PPD	C_44_H_74_O_14_	8.12	[M+HCOO]^−^	871.5055	871.5039	−1.85	825.5031 [M−H]^−^; 783.4895 [M−H-Ac]^−^	[30]
17	Vinaginsenoside R_2_	PPT	C_44_H_74_O_14_	8.58	[M+HCOO]^−^	871.5055	871.5039	−1.85	825.5031 [M−H]^−^; 783.4895 [M−H-Ac]^−^	[31]
18 *	Pseudoginsenoside F_11_	PPT	C_42_H_72_O_14_	9.25	[M+HCOO]^−^	845.4899	845.4913	1.70	799.4872 [M−H]^−^; 637.4328 [M−H-Glc]^−^; 475.3786 [M−H-2Glc]^−^	[30]
19	Ginsenoside Rf	PPT	C_42_H_72_O_14_	9.25	[M+HCOO]^−^	845.4899	845.4913	1.70	799.4872 [M−H]^−^; 637.4328 [M−H-Glc]^−^; 475.3786 [M−H-2Glc]^−^	[32]
20	Pseudoginsenoside Rt_4_	OT	C_36_H_62_O_10_	9.7	[M+HCOO]^−^	699.4320	699.4311	−1.22	653.434 [M−H]^−^	[31]
21	Ginsenoside Ra_0_	PPD	C_60_H_102_O_28_	9.93	[M+HCOO]^−^	1315.6534	1315.6572	2.88	1269.6510 [M−H]^−^,	[32]
22	Ginsenoside F_5_	PPD	C_41_H_70_O_13_	10.16	[M+HCOO]^−^	815.4793	815.479	−0.36	769.4743 [M−H]^−^; 637.4318 [M−H-Ara]^−^; 475.4197 [M−H-Ara-Glc]^−^	[30]
23 *	Ginsenoside Rg_2_	PPT	C_42_H_72_O_13_	11.15	[M+HCOO]^−^	829.4949	829.4953	0.43	783.4906 [M−H]^−^; 637.4308 [M−H-Rha]^−^; 475.3780 [M−H-Rha-Glc]^−^	[30]
24 *	Ginsenoside Rh_1_	PPT	C_36_H_62_O_9_	11.32	[M+HCOO]^−^	683.4370	683.4372	0.25	637.4366 [M−H]^−^; 475.3776 [M−H-Glc]^−^	[30]
25 *	Ginsenoside Rb_1_	PPD	C_54_H_92_O_23_	11.58	[M+HCOO]^−^	1153.6006	1153.6041	3.04	1107.5978 [M−H]^−^; 945.5436 [M−H-Glc]^−^; 783.4911 [M−H-2Glc]^−^; 621.4368 [M−H-3Glc]^−^	[30]
26 *	20R-Ginsenoside Rg_2_	PPT	C_42_H_72_O_13_	11.58	[M+HCOO]^−^	829.4949	829.4953	0.43	783.4906 [M−H]^−^; 637.4308 [M−H-Rha]^−^; 475.3780 [M−H-Rha-Glc]^−^	[30]
27 *	20R-Ginsenoside Rh_1_	PPT	C_36_H_62_O_9_	12.24	[M+HCOO]^−^	683.4370	683.4372	0.25	637.4366 [M−H]^−^; 475.3776 [M−H-Glc]^−^	[30]
28 *	Ginsenoside Ro	OA	C_48_H_76_O_19_	12.63	[M+HCOO]^−^	1001.4957	1001.4919	−3.82	955.4940 [M−H]^−^; 793.4398 [M−H-Glc]^−^	[30]
29 *	Ginsenoside Rc	PPD	C_53_H_90_O_22_	12.73	[M+HCOO]^−^	1123.5900	1123.5922	1.94	1077.5859 [M−H]^−^; 945.5434 [M−H-Araf]^−^; 783.4905 [M−H-Araf-Glc]^−^	[30]
30 *	Quinquenoside R_1_	PPD	C_56_H_94_O_24_	12.84	[M+HCOO]^−^	1195.6112	1195.6143	2.63	1149.6049 [M−H]^−^; 987.6059 [M−H-Glc]^−^	[31]
31	Quinquenoside R_1_ isomer	PPD	C_56_H_94_O_24_	13.06	[M+HCOO]^−^	1195.6112	1195.6143	2.63	1149.6049 [M−H]^−^; 987.6059 [M−H-Glc]^−^	[31]
32	Dimalonyl-ginsenoside Rd	PPD	C_54_H_86_O_24_	13.59	[M+HCOO]^−^	1163.5486	1163.5452	−2.88	1117.5467 [M−H]^−^; 955.5905 [M−H-Glc]^−^	[33]
33 *	Ginsenoside Rb_2_	PPD	C_53_H_90_O_22_	14.11	[M+HCOO]^−^	1123.5900	1123.5922	1.94	1077.5859 [M−H]^−^; 945.5456 [M−H-Arap]^−^; 783.4923 [M−H-Arap-Glc]^−^	[30]
34	Quinquenoside R_1_ isomer	PPD	C_56_H_94_O_24_	14.11	[M+HCOO]^−^	1195.6112	1195.6143	2.63	1149.6049 [M−H]^−^; 987.6059 [M−H-Glc]^−^	[31]
35 *	Ginsenoside Rb_3_	PPD	C_53_H_90_O_22_	14.61	[M+HCOO]^−^	1123.5900	1123.5922	1.94	1077.5856 [M−H]^−^; 945.5433 [M−H-Xyl]^−^; 783.4879 [M−H-Xyl-Glc]^−^;	[30]
36 *	Ginsenoside F_1_	PPT	C_36_H_62_O_9_	14.97	[M+HCOO]^−^	683.4370	683.4372	0.25	637.4366 [M−H]^−^; 475.3776 [M−H-Glc]^−^	[30]
37	Dimalonyl-ginsenoside Rd isomer	PPD	C_54_H_86_O_24_	15.03	[M+HCOO]^−^	1163.5486	1163.5452	−2.88	1117.5467 [M−H]^−^	[33]
38	Dimalonyl-ginsenoside Rd isomer	PPD	C_54_H_86_O_24_	15.7	[M+HCOO]^−^	1163.5486	1163.5452	−2.88	1117.5467 [M−H]^−^	[33]
39	Quinquenoside R_1_ isomer	PPD	C_56_H_94_O_24_	16.12	[M+HCOO]^−^	1195.6112	1195.6143	2.63	1149.6049 [M−H]^−^; 987.6059 [M−H-Glc]^−^	[31]
40	Dimalonyl-ginsenoside Rd isomer	PPD	C_54_H_86_O_24_	16.52	[M+HCOO]^−^	1163.5486	1163.5452	−2.88	1117.5467 [M−H]^−^	[33]
41 *	Ginsenoside Rd	PPD	C_48_H_82_O_18_	17.57	[M+HCOO]^−^	991.5478	991.5468	−0.98	945.5476 [M−H]^−^; 783.4890 [M−H-Glc]^−^; 621.4506 [M−H-Glc-Glc]^−^	[30]
42	Pseudoginsenoside Rc_1_	PPD	C_51_H_84_O_21_	18.81	[M+HCOO]^−^	1033.5583	1033.5546	−3.61	987.5548 [M−H]^−^	[32]
43	Pseudoginsenoside Rc_1_ isomer	PPD	C_51_H_84_O_21_	19.61	[M+HCOO]^−^	1033.5583	1033.5546	−3.61	987.5548 [M−H]^−^	[32]
44	Pseudoginsenoside Rc_1_ isomer	PPD	C_51_H_84_O_21_	20.18	[M+HCOO]^−^	1033.5583	1033.5546	−3.61	987.5548 [M−H]^−^	[32]
45	Gypenoside XVII	PPD	C_48_H_82_O_18_	21.56	[M+HCOO]^−^	991.5478	991.5468	−0.98	945.5476 [M−H]^−^; 783.4890 [M−H-Glc]^−^	[30]
46	Pseudoginsenoside Rc_1_ isomer	PPD	C_51_H_84_O_21_	21.92	[M+HCOO]^−^	1033.5583	1033.5546	−3.61	987.5548 [M−H]^−^	[32]
47 *	Notoginsenoside Fe	PPD	C_47_H_80_O_17_	23.86	[M+HCOO]^−^	961.5372	961.536	−1.25	915.5360 [M−H]^−^	[30]
48	Quinquenoside III	PPD	C_50_H_84_O_19_	24.03	[M+HCOO]^−^	1033.5583	1033.5587	0.36	987.5533 [M−H]^−^	[32]
49	Vinaginsenoside R_18_	PPD	C_47_H_80_O_17_	24.28	[M+HCOO]^−^	961.5372	961.535	−2.28	915.5329 [M−H]-; 783.4910[M−H-Xyl]-; 621.4327[M−H-Xyl-Glc]-	[30]
50	Ginsenoside Rk_3_	PPT	C_36_H_60_O_8_	24.66	[M+HCOO]^−^	665.4265	665.4271	0.95	619.4237 [M−H]^−^	[30]
51 *	Ginsenoside F_2_	PPD	C_42_H_72_O_13_	25.18	[M+HCOO]^−^	829.4949	829.4953	0.43	783.4906 [M−H]^−^	[30]
52 *	Ginsenoside Rg_3_	PPD	C_42_H_72_O_13_	25.68	[M+HCOO]^−^	829.4949	829.4953	0.43	783.4906 [M−H]^−^; 621.4308 [M−H-Glc]^−^	[30]
53	20R-Ginsenoside Rg_3_	PPD	C_42_H_72_O_13_	25.68	[M+HCOO]^−^	829.4949	829.4953	0.43	783.4906 [M−H]^−^; 621.4308 [M−H-Glc]^−^	[30]
54	Ginsenoside Rk_1_	PPD	C_42_H_70_O_12_	27.31	[M+HCOO]^−^	811.4844	811.4831	−1.58	765.4786 [M−H]^−^	[30]
55	Ginsenoside Rk_2_	PPD	C_36_H_60_O_7_	27.45	[M+HCOO]^−^	649.4316	649.4334	2.85	603.4353 [M−H]^−^	[34]

* Indicates that the component has been confirmed by comparison with reference standards.

## Data Availability

The original contributions presented in this study are included in the article/supplementary material. Further inquiries can be directed to the corresponding authors.

## Supplementary Materials

The following supporting information can be downloaded at: https://www.mdpi.com/article/10.3390/molecules31101570/s1. Figure S1: The extracted ion chromatogram of the target analytes in mixed standard solution; Figure S2: Heatmap analysis of the content determination results of saponin components in different parts of *P. vietnamensis*.; Table S1: Fourteen saponin components detected only in some sample groups; Table S2: Optimization of mass spectrometry parameters for target analytes; Table S3: Method validation results including the linearity, limit of detection (LOD), limit of quantification (LOQ), precision, recovery, repeatability, and stability; Table S4: Quantitative determination results of 21 saponin components in different *Panax* samples; Table S5: The sample information of plant materials of *Panax* species; Table S6: Detailed information on reference standards of saponins.
