# Supplementary material for: Comparative Analysis of the Ginsenosides in Panax vietnamensis and Three Panax Species
Source: Molecules. 2026 May 8;31(10):1570. doi: 10.3390/molecules31101570 (PMC13209941; doi:10.3390/molecules31101570)
Supplement: Supplementary file 1 [file molecules-31-01570-s001.zip › Supplementary Figure S2.pdf]

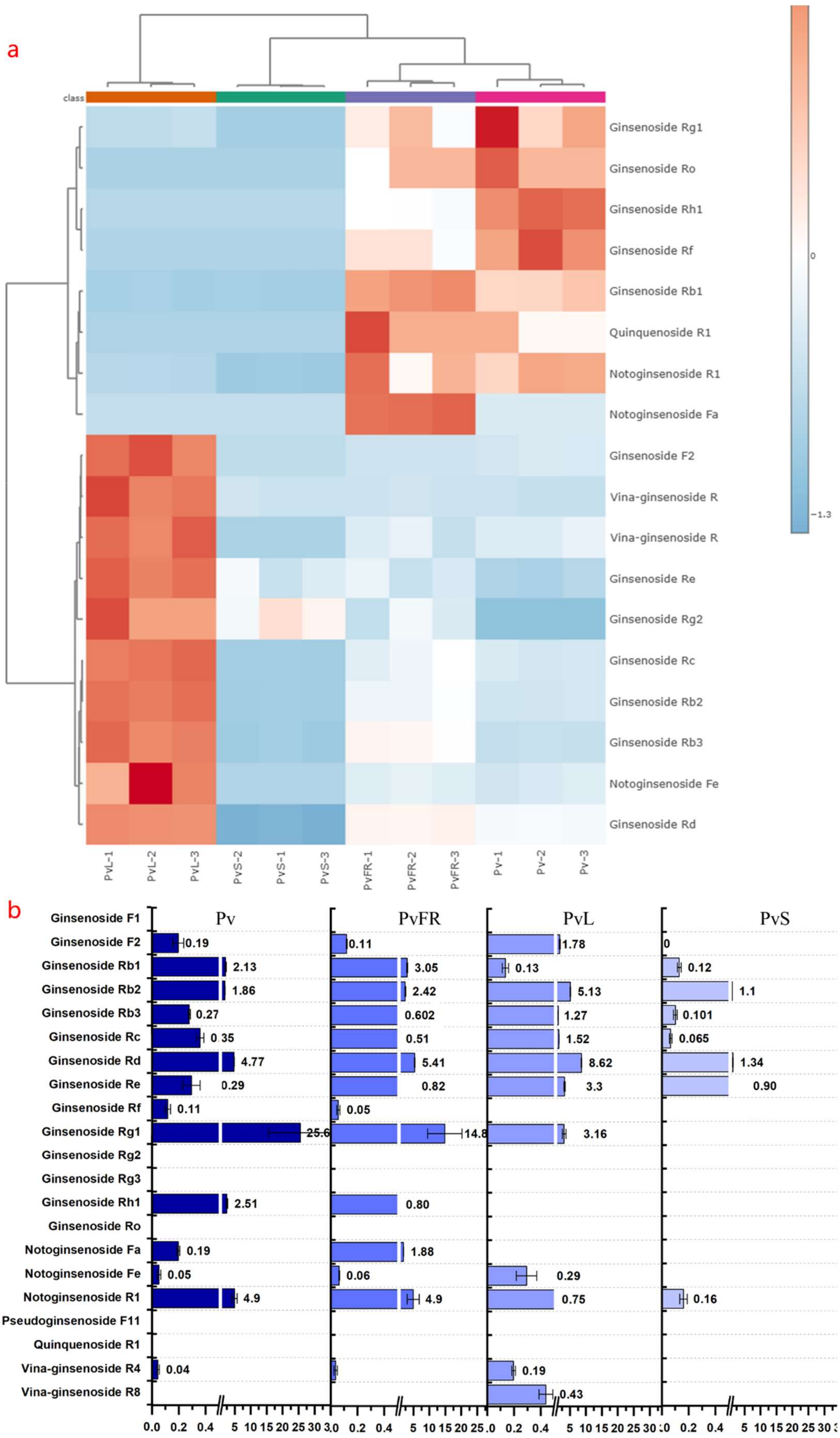

Figure S2. Heatmap analysis of the content determination results of saponin components in different parts of *P. vietnamensis*. The cluster heatmap (a), the bar chart of contents (b). Pv, *Panax vietnamensis* taproots; PvFR, *Panax vietnamensis* fibrous roots; PvL, *Panax vietnamensis* leaves; PvS, *Panax vietnamensis* stems;
