# Supplementary figures and images for: Comparative Analysis of the Ginsenosides in Panax vietnamensis and Three Panax Species
Source: Molecules. 2026 May 8;31(10):1570. doi: 10.3390/molecules31101570 (PMC13209941; doi:10.3390/molecules31101570)

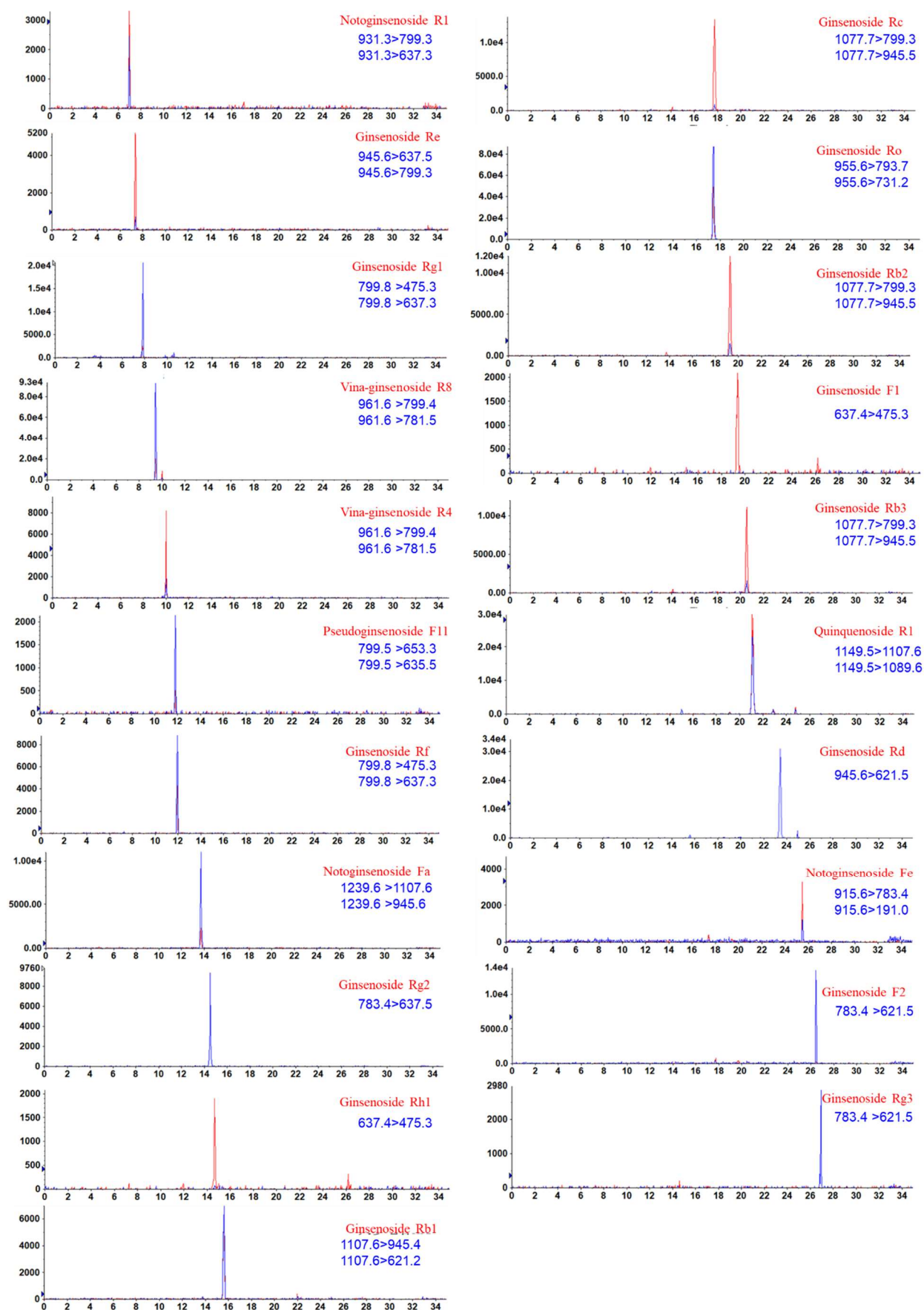

Figure S1. The extracted ion chromatogram of the target analytes in mixed standard solution.

Supplement: Supplementary file 1 [file molecules-31-01570-s001.zip › Supplementary Figure S1.pdf]
